# Acoustofluidics-enhanced biosensing with simultaneously high sensitivity and speed

**DOI:** 10.1038/s41378-024-00731-3

**Published:** 2024-06-29

**Authors:** Yuang Li, Yang Zhao, Yang Yang, Wenchang Zhang, Yun Zhang, Sheng Sun, Lingqian Zhang, Mingxiao Li, Hang Gao, Chengjun Huang

**Affiliations:** 1 Institute of Microelectronics of the Chinese Academy of Sciences, Beijing, 100029 P. R. China; 2 University of Chinese Academy of Sciences, Beijing, 101408 P. R. China; 3 Department of Medicine, Brigham and Women’s Hospital, Harvard Medical School, Boston, MA 02115 USA

**Keywords:** Biosensors, Microfluidics

## Abstract

Simultaneously achieving high sensitivity and detection speed with traditional solid-state biosensors is usually limited since the target molecules must passively diffuse to the sensor surface before they can be detected. Microfluidic techniques have been applied to shorten the diffusion time by continuously moving molecules through the biosensing regions. However, the binding efficiencies of the biomolecules are still limited by the inherent laminar flow inside microscale channels. In this study, focused traveling surface acoustic waves were directed into an acoustic microfluidic chip, which could continuously enrich the target molecules into a constriction zone for immediate detection of the immune reactions, thus significantly improving the detection sensitivity and speed. To demonstrate the enhancement of biosensing, we first developed an acoustic microfluidic chip integrated with a focused interdigital transducer; this transducer had the ability to capture more than 91% of passed microbeads. Subsequently, polystyrene microbeads were pre-captured with human IgG molecules at different concentrations and loaded for detection on the chip. As representative results, ~0.63, 2.62, 11.78, and 19.75 seconds were needed to accumulate significant numbers of microbeads pre-captured with human IgG molecules at concentrations of 100, 10, 1, and 0.1 ng/mL (~0.7 pM), respectively; this process was faster than the other methods at the hour level and more sensitive than the other methods at the nanomolar level. Our results indicated that the proposed method could significantly improve both the sensitivity and speed, revealing the importance of selective enrichment strategies for rapid biosensing of rare molecules.

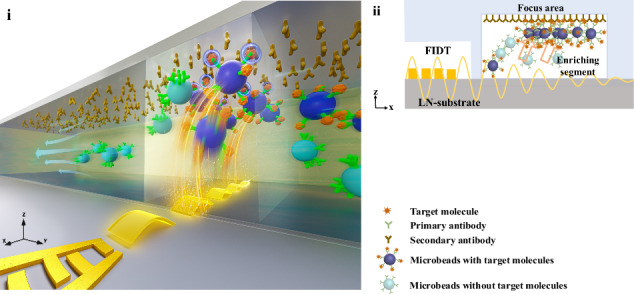

## Introduction

Biomolecular marker detection technologies are widely used in clinical testing (e.g., influenza and other infectious diseases, pregnancy testing, etc.) and frontier scientific research (e.g., tumor marker detection, exosome detection, etc.)^1–3^. In these applications, biosensors primarily utilize soluble molecule diffusion to perform reactions for detection^4,5^. Due to the uncertainty of the passive diffusion process, the difficulty of collecting target molecules in the local biosensing region is a cross-scale challenge, such as trying to observe the taste change on a small landing stage located in a one-kilometer-wide pond after a drop of wine falls into the pond^6^. Thus, an excessive incubation time of hours needs to be consumed in every step for two or more steps for biosensing in traditional methods. Even worse, these methods often cannot collect enough target molecules into the biosensing region by passive diffusion, resulting in “false-negatives” at low concentrations^7,8^.

The direct approach to improve sensitivity is to utilize physical or chemical effects to increase the signal gains^9–11^; these effects include surface electrochemical effects^12,13^ and surface electric field-enhanced ion-sensitive transistors^6,14^. surface-enhanced Raman scattering^15,16^, local field enhancement by nanoparticles^17,18^, or even a molecular electromechanical system (MolEMS)^19^. However, the approach that only pursues signal amplification may result in “false-negatives” or “false-positives” caused by insufficient target molecules being captured or noise increasing, respectively, leading to baseline drifting.

Another approach to improve sensitivity is the introduction of external physical fields, such as charge adsorptions^20,21^, magnetic bead adsorption^22,23^, and acoustic surface wave effects^24,25^, to facilitate target molecule enrichment. However, “false-positives” resulting from nonspecific binding must be considered in those enrichments, and cleaning nonspecific binding is essential^26–28^. Moreover, due to the hindering effect caused by the nonspecific binding of the invalid samples, the weak signals of rare molecules would be swamped in these enrichment methods.

To collect more sufficient target molecules for biosensing regions and simultaneously wash away nonspecific binding molecules, researchers have developed microfluidic techniques to allow the target molecules in the liquid to continuously flow through microchannels and pass through the local biosensing region in the microchannel to be detected^29^. However, the inherent laminar flow inside the microscale channel is the main obstacle to improving the binding efficiency of biomolecules.

**Fig. 1:**
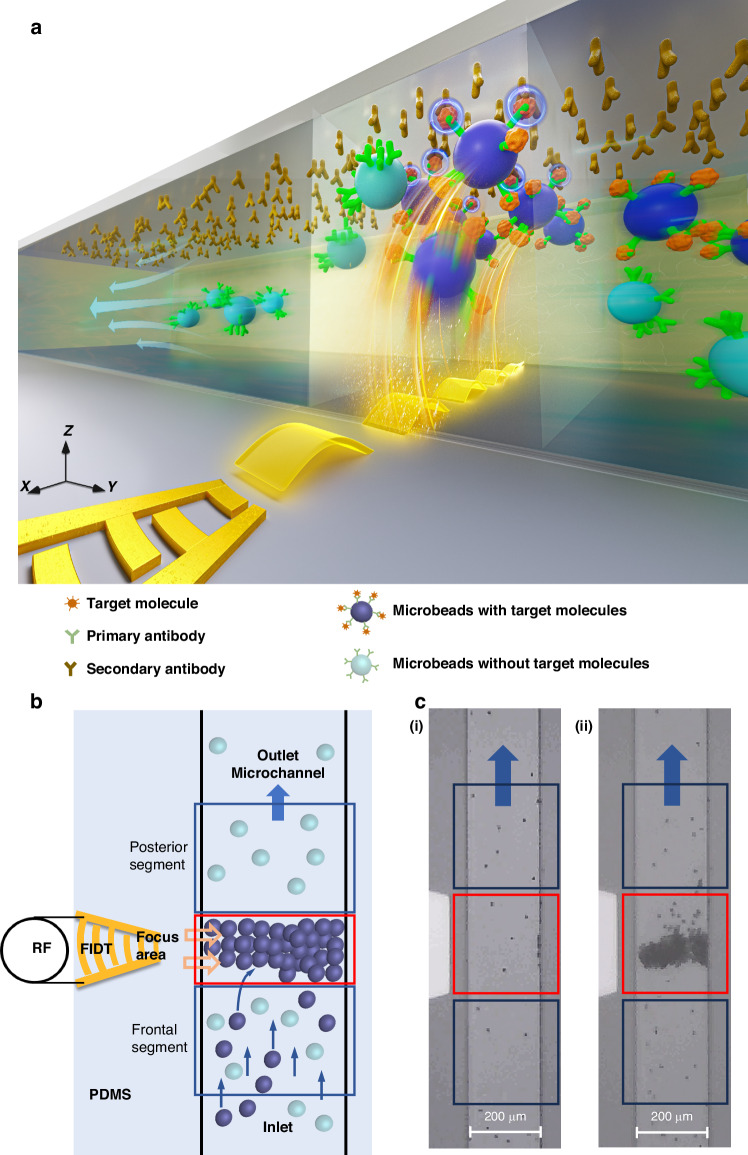
Schematic plots of the acoustofluidics-enhanced biosensing method and photos of the typical contrast results. **a** Schematic of acoustofluidic-enhanced biosensing, which enables continuous specific enrichment and detection in microchannels. **b** The top view shows the enrichment and detection process in detail. The red area represents the focus area for the specific enrichment of microbeads pre-captured with target molecules. **c** Detailed photos of the specific enriched microbeads carrying target molecules without (i) or with (ii) acoustofluidics enhancement

Here, we propose a new approach to improve biosensing sensitivity and detection speed by actively enriching the target molecules with an acoustic microfluidic-enhanced method, as shown in Fig. 1a. The acoustic force exploited by acoustic microfluidics has the advantages of high-precision manipulation of microparticles and insensitivity to fluid and particle composition^30,31^. To address the challenge of uncertainty in the passive diffusion process, we mainly utilized the acoustic force generated by the focused traveling surface acoustic waves (FTSAWs) to rapidly enrich the microbeads; these microbeads were pre-captured with rare target molecules from milliliter samples to specifically bind to the biosensing region (Fig. 1b) to directly indicate the biomolecule content by counting the collected microbeads (Fig. 1c).

## Results and discussion

### Experimental setup and analysis

**Fig. 2:**
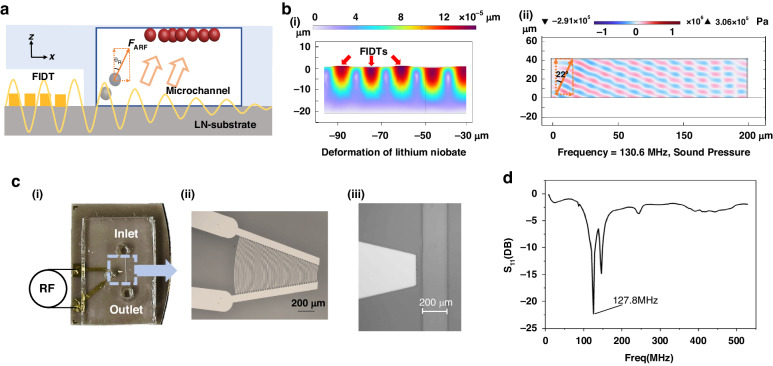
Experimental design, modeling, and setup. **a** X-Z plane showing the enrichment of PS microbeads in detail. **b** Simulation analysis of the device. (i) Deformation of lithium niobate. (ii) Simulation of the sound pressure in the microchannel. **c** Morphological characteristics of the device. (i) Picture of the device. (ii) Micrographs of the FIDT and (iii) microchannel. **d** Plot of the S_11_ parameters of the FIDT based on vector mesh analyzer measurements

As shown in Fig. 2a, we proposed using the acoustic radiation force to push almost all of the passed microbeads to the top surface of the focus area, where the microbeads carrying the target molecules would be captured, while the microbeads without target molecules would be washed away. FTSAWs are excited by the resonance of focused interdigital transducers (FIDTs) and propagate as leaky surface acoustic waves (LSAWs) enter the fluid in the microchannel; here, the acoustic fields exert acoustic radiation forces on the particles. In theory, the FTSAW propagated toward the PDMS microchannel and radiated into the fluid at a Rayleigh angle θ_R_ of approximately 22°, as calculated by Eq. (1):

1 $${{\rm{\theta }}}_{{\rm{R}}}={\sin }^{-1}({{\rm{C}}}_{{\rm{f}}}/{{\rm{C}}}_{{\rm{s}}})$$

where *c*_f_ and *c*_s_ are the speed of sound in the fluid and on the LiNbO_3_ substrate, respectively, with *c*_f_ = 1495 m/s and *c*_s_ = 3978 m/s.

For visualization, we carried out a modeling simulation. As shown in Fig. 2b, the simulation results showed that the FIDTs could cause significant deformations and form surface acoustic waves that propagate forward on the surface of the LN substrate under the influence of RF power (Fig. 2b-i). The simulation revealed the longitudinal radiation force of the acoustic radiation force in the propagation process, as shown in Fig. 2b-ii. Moreover, the resonance frequency generated by the simulation was ~130.6 MHz, and the acoustic radiation force had an evident longitudinal distribution. The acoustic field distribution had a diffraction angle of ~22°, indicating that the acoustic radiation forces exerted by the LSAW could be applied to push the microbeads to the top surface of the microchannel.

When the microbeads for the channel flow through the focus area, the microbeads are pushed away from the acoustic source and gather at the top of the channel by the force of acoustic radiation. Since the acoustic radiation force is sensitive to the polystyrene (PS) microbead size, a dimensionless factor *κ* is defined to indirectly evaluate the acoustic radiation force acting on the PS microbeads in the FTSAW field. The value of *κ* is determined by the particle diameter and the acoustic frequency with the relation in Eq. (2)^32^:

2 $${\rm{\kappa }}={\rm{\pi }}{\rm{df}}/{{\rm{c}}}_{{\rm{f}}}$$

where *d* is the microbead size and f is the resonance frequency. When *κ* is >1, the microbeads move in response to the acoustic radiation force. According to Eq. (2), the threshold size of the microbeads subjected to acoustic forces is 3.8 µm. When the size of the microbeads is larger than 3.8 μm, directional movement occurs due to the acoustic force; however, when the size of the microbeads is smaller than 3.8 μm, the microbeads are subjected only to the laminar flow of the fluid in the channel without being affected by the acoustic force. Microbeads of 3 μm and 7 μm in size were used for experimental validation.

Photos of the experimental setup are shown in Fig. 2c. Figure 2c-i–iii show the fabricated acoustic microfluidic chip, the FIDT electrode, and the microchannel, respectively.

Moreover, we characterized the S_11_ curve of the FIDT and found that the FIDT had a resonant frequency of 127.8 MHz, as shown in Fig. 2d. The deviation from the simulation results was potentially caused by the lattice changes in the actual lithium niobate substrate used due to the blackening process. The second drop was at 147.4 MHz. However, we found that the FIDT could not excite an effective resonance to generate an acoustic force to drive the microbeads at this frequency. Therefore, in subsequent experiments, we used the 127.8 MHz RF signal as the excitation signal for the FIDT.

### Microbead enrichment

**Fig. 3:**
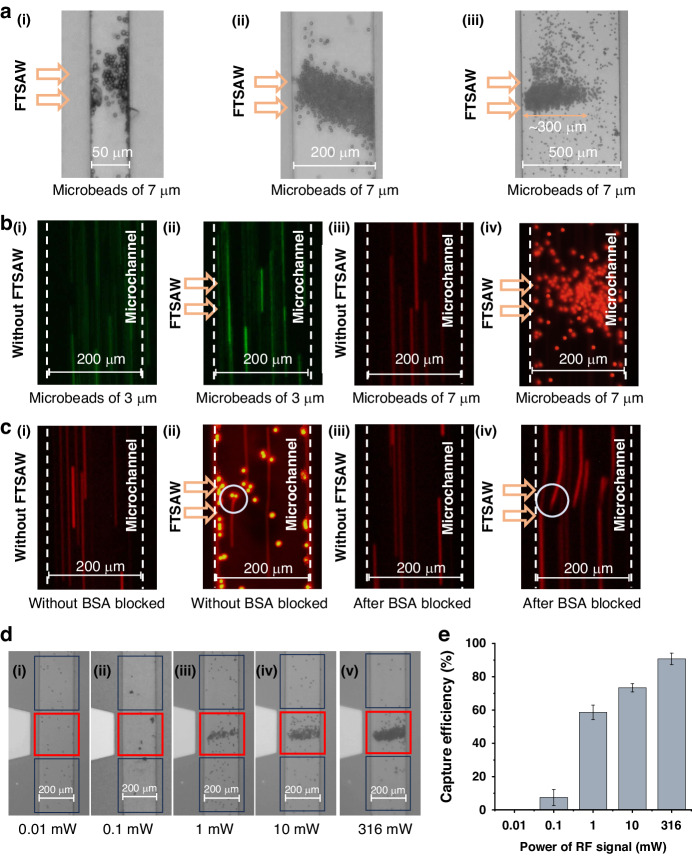
Characterization of the critical parameters of the microbeads and the microfluidic device. **a** Micrographs of microbead enrichment at different channel widths. The widths are (i) 50 µm, (ii) 200 µm, and (iii) 500 µm. **b** Comparative plots of the results for 3 μm (i, ii) and 7 μm (iii, iv) fluorescent microbeads. **c** Flow trajectories of 7 µm fluorescent microbeads before (i, ii) and after (iii, iv) BSA blocking. **d** Micrographs and **e** Statistical plots of 7 µm microbead enrichment efficiency using different intensities of RF signals

The enrichment results of the 7 µm microbeads under the same test conditions in microchannels of different widths are shown in Fig. 3. Figure 3a shows the enrichment of microbeads when different microchannel widths are used. As shown in Fig. 3a-i, when the channel width is 50 µm, most of the enrichment microbeads appear far from the sound source, which is attributed to the Rayleigh angle *θ*_R_ of the acoustic radiation force during diffraction into the channel; this results in a smaller effective area for the enrichment of the microbeads. When the width is extended to 500 μm, as shown in Fig. 3a-iii, the FTSAW is attenuated along the propagating path and cannot efficiently enrich microbeads at a position (~300 µm) away from the sound source. For a width of 200 µm, the enrichment effect is shown in Fig. 3a-ii; here, the top area of the channel can be fully used for sample enrichment, indicating that a width of 200 µm is optimal.

To verify the enrichment effect of acoustic force on microbeads, we selected fluorescent microbeads with diameters of 3 and 7 μm, and the results are shown in Fig. 3b. The results showed that acoustic forces were size-selective for microbeads. As shown in Fig. 3b-i and ii, regardless of whether the FTSAWs were excited, the motion trajectories of the 3 µm microbeads did not undergo a lateral shift. In contrast, the motion trajectories of the 7 µm microbeads did not laterally shift without FTSAW (Fig. 3b-iii) but were captured immediately on the top surface of the microchannel when the FTSAW was applied (Fig. 3b-iv), indicating that 7 µm microbeads were appropriate for active enrichment.

The fluorescence trajectories of the microbeads are shown in Fig. 3c. White circles are used to highlight variations in the trajectories of the microbeads. Figure 3c-i shows that no lateral shift can be observed when the microbeads pass through the microchannel without BSA blocking and without FTSAW propagation. As shown in Fig. 3c-ii, significant lateral trajectories of the passing microbeads and the high spots of the captured microbeads can be observed when the microbeads pass the microchannel without BSA blocking but with FTSAW propagating. Figure 3c-iii shows no lateral shift can be observed when microbeads pass the microchannel with BSA blocking and without FTSAW propagating. As shown in Fig. 3c-iv, significant lateral trajectories can be observed, but no microbeads are captured when the microbeads pass through the microchannel with BSA blocking and FTSAW propagation. The comparative results show that the molecular force that is potentially caused by the functional group (-COOH) on the top surface of the microchannel plays a crucial role in binding acoustic force-driven microbeads to channels to achieve enrichment. The results also show that BSA blocking can prevent nonspecific binding.

Figure 3d shows the microbead enrichment efficiencies using different intensities of RF signal sources. Figure 3d-i shows that the FTSAW fails to capture the microbeads when the power at the resonance frequency is too low. Figure 3d-ii to v show that the number of captured microbeads increased as the intensity of the RF signal source increases. When the applied power of the FIDT is -10 dBm (0.1 mW), the microbeads achieve directional capture under the action of the FTSAW despite the low capture efficiency; thus, we consider the power at this point as the threshold power for capturing the microbeads. Figure 3e shows that the enrichment efficiencies are approximately 0, 7.5%, 60%, 75%, and 91% when the RF signal intensities are −20 dBm (0.01 mW), -10 dBm (0.1 mW), 0 dBm (1 mW), 10 dBm (10 mW), and 25 dBm (316 mW), respectively; these results indicate that almost all of the microbeads can be captured when a sufficient RF signal strength is applied as the driving source.

### Biosensing based on antibody-antigen binding

**Fig. 4:**
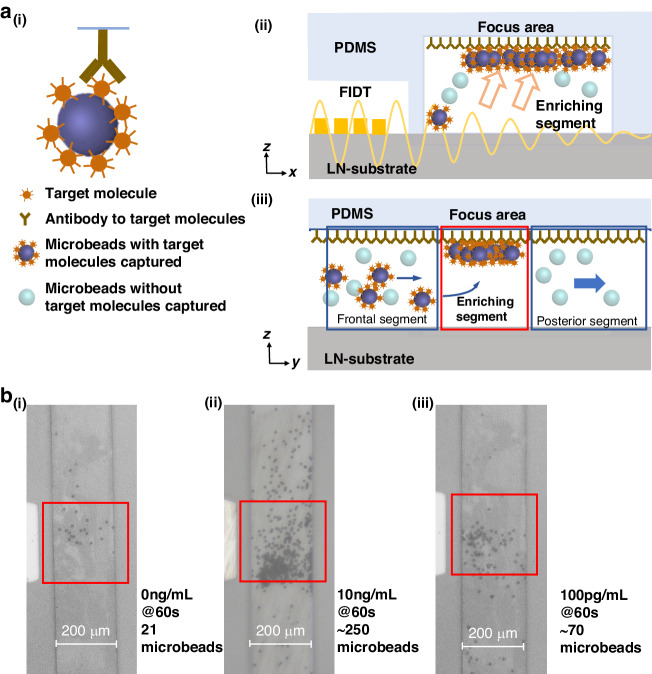
Biosensing experiment based on antibody-antigen binding. **a** Detailed detection process (i) and the x-z plane (ii) and y-z plane (iii) show enrichment. **b** Microscope images of the captured 7 µm microbeads when the concentrations of the target molecules were 0 (i), 10 ng/mL (ii), and 100 pg/mL (iii)

Since molecular forces can be exploited to bind microbeads to the top surface of microchannels in the focus area, the antibody-antigen binding is used to capture and enrich microbeads for specific biosensing (Fig. 4a). As shown in Fig. 4a-i and ii, driven by the acoustic radiation force, the microbeads, which nonspecifically pre-adsorbed the human IgG molecules from the samples, were pushed to undergo immune binding on the top surface with a specific antibody coating, achieving specific enrichment as the biosensing result in the focus area (Fig. 4a-iii).

Figure 4b shows micrographs of microchannels with microbeads captured for different target molecule concentrations. Figure 4b-i shows that ~21 microbeads were nonspecifically captured for 60 seconds in the focus area (red box); these results indicated that the baseline number was ~21. As shown in Fig. 4b-ii, at 10 ng/mL, ~250 microbeads were captured in 60 s; this value was greater than the baseline number for the control group. As shown in Fig. 4b-iii, at 100 pg/mL, ~70 microbeads were captured; this value was still higher than the baseline number, indicating that the limit of detection (LOD) was lower than 100 pg/mL. These preliminary results showed that the proposed acoustic microfluidic-enhanced biosensing method utilizing antibody-antigen binding could simultaneously improve the sensitivity and speed. Moreover, the biosensing results could be easily read without complex detection equipment.

**Fig. 5:**
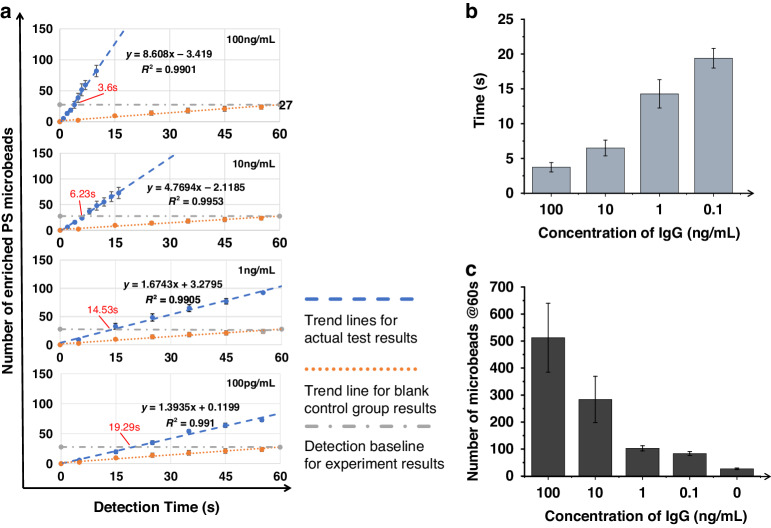
Characterization of biosensing performance based on antibody-antigen binding. **a** Plots of the number of enriched microbeads at different concentrations to characterize the detection time versus time. Statistical plots of the **b** biosensing time versus the molecule concentration and **c** total number of collected microbeads in 60 seconds for the different molecule concentrations

Figure 5 shows the statistical results from the biosensing experiment based on antibody-antigen binding. As shown in Fig. 5a, the mean number of enriched microbeads increased linearly over time for all groups with five different target molecule concentrations. The baseline number was statistically 27; thus, 27 microbeads were captured for 60 seconds in the control group. When the target molecule concentration was 100 ng/mL, ~3.6 s were needed to enrich a significant number of microbeads, and when the concentration was as low as 100 pg/mL, the biosensing time was only 19.29 s; these results show the fast biosensing speed attained with our method. The biosensing time, defined as the time spent enriching a significant number of microbeads in every experiment, was measured at different concentrations. As shown in Fig. 5b, the biosensing times were 3.75 ± 0.68 s, 6.5 ± 1.13 s, 14.28 ± 2.03 s, and 19.4 ± 1.42 s for molecular concentrations of 100, 10, 1, and 0.1 ng/mL, respectively. As shown in Fig. 5c, the number of enriched microbeads at 60 s was 512 ± 127, 284 ± 86, 103 ± 10, 83 ± 8, and 27 ± 3 for molecular concentrations of 100, 10, 1, 0.1, and 0 ng/mL, respectively, indicating that the potential LOD of the device was lower than 100 pg/mL. In summary, the results show that the proposed acoustic microfluidic method has the potential for rapid and highly sensitive biosensing based on antibody-antigen binding.

### Sandwiching biosensing

**Fig. 6:**
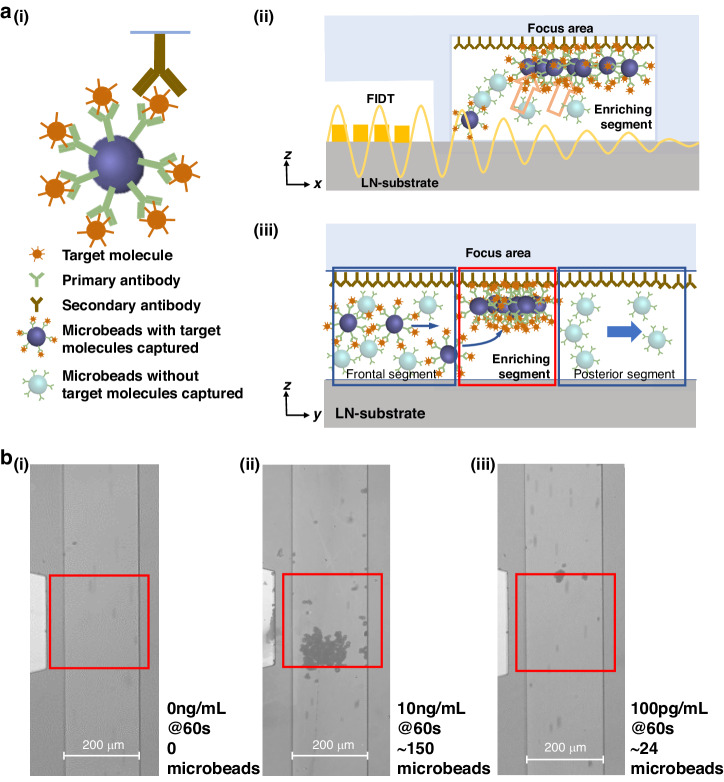
Biosensing experiment based on the sandwiching reaction. **a** Detailed detection process (i) and the x-z plane (ii) and y-z plane (iii) show enrichment. **b** Microscope images of the captured 7 µm microbeads at concentrations of 0 (i), 10 ng/mL (ii), and 100 pg/mL (iii)

To further improve the specificity, we used a sandwiching biosensing method with enhanced FTSAWs to detect the target molecules. The anti-human IgG FITC was surface-modified on the top surface of the microchannel, and the goat anti-human IgG (Fab specific) was surface-modified on the surface of the PS microbeads. Then, the microbeads were added to the microchannels after capturing the target molecules from every 1 mL sample. When flowing in the microchannel, the microbeads were moved and underwent the sandwiching reaction on the top surface of the focus area under acoustic force (Fig. 6a).

As shown in Fig. 6b, the raw results could be directly read, and the number of microbeads in the control group was significantly reduced to almost zero (Fig. 6b-i); these results indicated that the sandwiching biosensing method could improve the accuracy by preventing nonspecific binding. The results also showed that the microbeads were significantly enriched when the molecule concentration was 10 ng/mL (Fig. 6b-ii), and distinct clusters of microbeads could be found in the sensing region; thus, the biosensing results were still visible when the molecule concentration was as low as 100 pg/mL (Fig. 6b-iii).

**Fig. 7:**
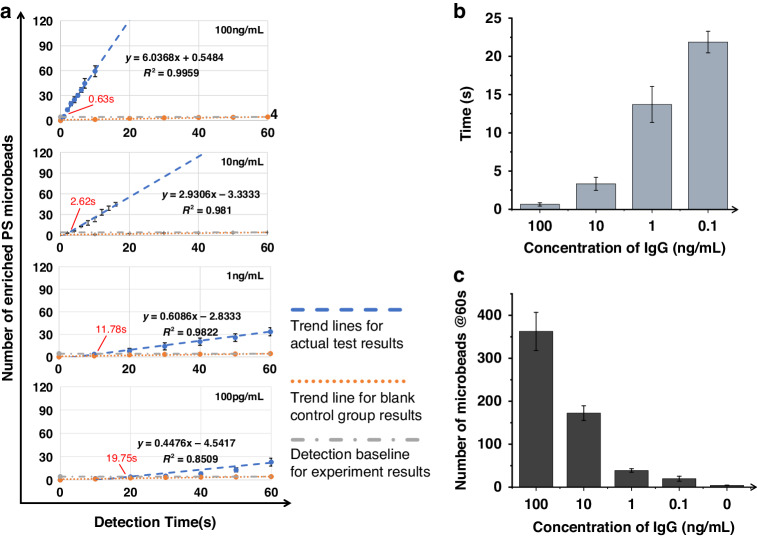
Characterization of biosensing performance based on the sandwiching reaction. **a** Plots of the number of enriched microbeads at different concentrations to characterize the detection time versus time. Statistical plots of the **b** biosensing time versus the molecule concentration and **c** total number of collected microbeads in 60 seconds for the different molecule concentrations

As shown in Fig. 7a, the number of active enriched microbeads linearly increased over time for different concentrations at 60 seconds. When the concentration of the target molecule was 100 ng/mL, ~0.63 s was needed to collect a significant number (>baseline number) of microbeads; moreover, when the concentration of the target molecule was 100 pg/mL, ~19.75 s was needed to collect the microbeads, and this was still considered fast. As shown in Fig. 7b, the biosensing times were 0.64 ± 0.22 s, 3.32 ± 0.85 s, 13.7 ± 2.35 s, and 21.87 ± 1.41 s for molecular concentrations of 100, 10, 1, and 0.1 ng/mL, respectively. As shown in Fig. 7c, the number of enriched microbeads after 60 s was 363 ± 44, 172 ± 17, 39 ± 4, 20 ± 6, and 4 ± 1 for molecular concentrations of 100, 10, 1, 0.1, and 0 ng/mL, respectively. Figure 7c shows that the number of enriched microbeads after 60 s decreased as the concentration decreased to 100 pg/mL; additionally, even when the target molecule concentration was 100 pg/mL, the number of enriched microbeads was still greater than that in the control group; these results indicated that the proposed method could be applied for rarer molecules. Furthermore, compared to that of the control group, as shown in Fig. 5c, the number of microbeads in the control group was significantly lower, indicating that the sandwiching reaction could reduce the baseline drift caused by nonspecific binding.

Based on the results from these experiments, the molecular binding probability is significantly increased by active enrichment with the acoustic force, and the assay reduces both the biosensing time and the LOD of traditional methods. traditional methods would typically need long times to complete their analyses, and their LODs are still at the nanomolar level. The use of newly developed biomolecule enrichment methods, such as quantum dots, for the enhanced electrochemical detection of human IgG (1.7 pM, 50 min) has been explored^33^. Sensitive sandwich ELISA is based on a gold nanoparticle layer (2 ng/mL)^34^, angular plasmonic biochemical sensors (9.93 pM, 20 min)^35^, and a duplex electrochemical microfluidic sensor (15 ng/mL, 7 min)^36^; however, our proposed active targeting method still has advantages in biosensing speed and sensitivity.

In previous studies, the magnetic approach (magnetic field + magnetic beads) and many other external forces/fields, such as optical tweezers and dielectrophoresis, have been used for biosensing and biodetection, in which the magnetic approach has been widely used for selectively enriching magnetic bead-labeled target molecules. For example, Ruiz-Vega et al.^37^ used magnetic beads with a magnetic field, electrodes, and paper-based microfluidics to successfully detect malaria enzymes in whole blood within 20 minutes. Fortunati et al.^38^ used magnetic beads as versatile tools for electrochemical biosensing platforms in point‐of‐care testing. However, in these studies, the integration of the generator of the external force on the microdevice and the precise control of the force were still challenging^39^. In this study, our proposed acoustic-fluidic method showed the advantages of size selectivity and easy integration and was more suitable for microfluidic applications.

Moreover, the results from this proposed method could be directly read without transducing by high-precision signal acquisition equipment or devices, in contrast to other sophisticated biosensing methods^40–43^.

## Conclusion

To address the challenge of simultaneously high sensitivity and speed for rare molecule detection caused by passive diffusion and disordered binding, we proposed an acoustic microfluidic method that enabled the specific enrichment of microbead-carried molecules. By exploiting the ability of the FTSAW to perform precise microparticle manipulation, we demonstrated that the developed chip could specifically bind a significant number of microbeads pre-captured with human IgG molecules at concentrations ranging from low to 100 pg/mL in approximately 22 s. Our results indicated the importance of specific enrichment for detecting rare molecules. Furthermore, this demonstration also revealed the potential of acoustic microfluidic techniques for overcoming the cross-scale challenges of many biosensors by precisely manipulating multiple microparticles to rapidly transport biomolecules diffusing in a milliliter volume to be enriched to a nanoliter volume for sensing.

## Materials and methods

### Reagents

The relevant materials used for immunoassay and modified processing in the experiment included BSA (Thermo Fisher, USA, 37525), phosphate-buffered saline (GIBCO, Life Technologies Corp., USA), MES (Thermo Fisher, USA, 28390), 1-(3-dimethylaminopropyl)3-ethylcarbodiimide hydrochloride (Thermo Fisher, USA, 22980), and N-hydroxysuccinimide (Thermo Fisher, USA, 24500). 3-Aminopropyl triethoxysilane (Sigma‒Aldrich, DE, A3648) and glutaraldehyde aqueous solutions (Sigma‒Aldrich, DE, G5882) were used to modify the surface of the top surface of the microchannels. Human IgG (Sigma‒Aldrich, DE, I4506), anti-human IgG FITC (Sigma‒Aldrich, DE, F9512), and goat anti-human IgG (Fab specific) (Sigma‒Aldrich, DE, F5512) were used for biomolecular enrichment and detection. The materials used for device manufacture included SU-8 photoresist (MicroChem Corp., USA) and 184 silicone elastomers (Dow Corning Corp., USA) for the microfluidic chips.

### Design, modeling, and fabrication

The microfluidic chip was bonded to a lithium niobate piezoelectric substrate and a PDMS cover. A FIDT was patterned on the substrate, while a through-channel and a FIDT cavity were molded under the PDMS cover. The FIDT cavity was designed to cover the FIDT to reduce the attenuation of the FTSAW before it entered the microchannel. In the FIDT design, we used sector comb electrodes to provide a focused acoustic surface wave in the focusing region to reduce the loss of acoustic energy during propagation^44^. The FIDT had 36 pairs of electrodes with a focus angle of 26 degrees to better minimize fork-finger electrode insertion loss^45,46^. The width and gap of the electrodes were chosen to be 7 μm.

During modeling, the electrostatics module, solid mechanics module, and multiphysics module (piezoelectric effect) were used in the vibration modeling, while the multiphysics module of the acoustics-structure boundary was used to introduce the SAWs in the surface of the piezoelectric layer (lithium niobate) to the liquid zone (water). The boundaries of the liquid zone were set as the perfect match layer. The acoustic pressure module was used to show the distribution of acoustic waves in the liquid zone. The electrodes were made of gold.

A 4″ single-polished 128° Y–X cut 500 μm thick blackened lithium niobate wafer was used as the piezoelectric substrate because of its sufficient strength, good electromechanical coupling, low body wave generation, and high surface acoustic wave velocity^47,48^. During fabrication, the mold of the PDMS cover was fabricated with SU-8 photoresist with a layer height of 40 µm. The FIDT gold electrodes (200 nm in height) were patterned on the bottom lithium niobate substrates via the conventional lift-off microfabrication procedure (Fig. S1). The conductive silver adhesive was used as a connecting layer between the FIDT and the ESG vector signal generator (Agilent, USA, E4438C). Before use, the S_11_ of the FIDT was tested using a vector network analyzer (R&S ZVL NETWORK ANALYZER, DE) to obtain the resonance frequency.

### Assessment of critical parameters

Three types of channels with widths of 50, 200, and 500 µm were fabricated to characterize the effective range of actuating ranges of FTSAWs to determine the appropriate size of the microchannel. The output power of the RF signal generator was set to 25 dBm (316 mW), and enrichment characterization was performed using microbeads with a diameter of 7 µm. The effect of channel width was analyzed by observing the number of enriched microbeads and the length of the microbead-enriched region.

Two types of microbeads (7 and 3 µm in diameter) were used to assess the enrichment effects of microbeads with different diameters. Fluorescent microbeads and long-exposure (0.1 s exposure time) fluorescence microphotography were used to photograph the flow trajectories of the fluorescent microbeads to characterize the movement of the PS microbeads and verify that the forces are more dominant in the channel height direction.

Five RF signal powers of −20 dBm (0.01 mW), −10 dBm (0.1 mW), 0 dBm (1 mW), 10 dBm (10 mW), and 25 dBm (316 mW) were applied to the FIDTs using the ESG vector signal generator to assess the enrichment effects under different RF signal driving strengths. The performance was assessed by accounting for the difference between the number of enriched and total microbeads, while the instantaneous number of microbeads enriched at a given time point was calculated by subtracting the number of microbeads in the frontal segment and the number of microbeads in the posterior segment.

Contrast experiments of microbead enrichment effects in the microchannel were performed without or with BSA blocking to assess the nonspecific enrichment effects of BSA blocking. Fluorescent microbeads and long-exposure fluorescence (0.1 s exposure time) microphotography were used to visually observe the flow trajectories of the fluorescent microbeads.

Moreover, the supporting information provides detailed methods and results for PS microbead and microchannel surface modifications.

### Biosensing based on immune binding

For biosensing the immune molecules, the microbeads and channel surfaces were first fluorescently modified using a fluorescent antibody, and the target molecules were characterized for successful modification based on whether they could be excited to fluoresce (Fig. S2). Before use, the microchannels were precoated with FITC-conjugated anti-human IgG.

In antigen-antibody immune binding-based experiments, 100,000 PS microbeads were added to 1 mL samples with IgG molecules at four concentrations (100, 10, 1, and 0.1 ng/mL), incubated for 2 hours and then added to the microchannel for enrichment for detection. Moreover, these experiments employed microbeads without target molecules as the control group.

In sandwiching immune binding-based experiments, anti-human IgG FITC is surface-modified on the surface of the channel, and goat anti-human IgG (Fab specific) is surface-modified on the surface of the PS microbeads. After being blocked by BSA, 100,000 PS microbeads were added to 1 mL samples with the IgG molecules at four concentrations (100, 10, 1, and 0.1 ng/mL), incubated for 2 hours, and then added to the microchannel to be enriched for detection. Moreover, these experiments employed microbeads without target molecules as the control group.

To characterize the detection performance of the assay, the number of microbeads adsorbed by the control group at the same time was used as the baseline of the experiment, and the number of microbeads during the enrichment process of the experimental group was recorded, plotted as a dot plot, and fitted to the data. The time at which the fitted straight line intersected the baseline was used as the detection time of the sample. Furthermore, the LOD is the lowest concentration at which the detection criteria can be achieved. Notably, this detection capability is not theoretical but practical. Finally, the number of microbeads enriched for detection in 60 s was counted and statistically analyzed with respect to the detected concentration, which was used to characterize the detection concentration capability of the proposed method.

### Data analysis

During the counting and fluorescence analysis of the PS microbeads, ImageJ software, as recommended by the National Institutes of Health, was used for counting or analyzing the microbeads on the bright-field and fluorescence images. All data in this study are expressed as the mean ± SEM from no less than three independent assays. All experiments were repeated at least three times.

### Supporting information

Fabrication and testing of FIDT; modification methods for the PS microbeads and microchannels; characterization and validation of surface modification methods.

### Supplementary information

Acoustofluidics-enhanced biosensing with simultaneously high sensitivity and speed
