# Supplementary material for: Acoustofluidics-enhanced biosensing with simultaneously high sensitivity and speed
Source: Microsyst Nanoeng. 2024 Jun 29;10:92. doi: 10.1038/s41378-024-00731-3 (PMC11217392; doi:10.1038/s41378-024-00731-3)
Supplement: Supplementary file 1 — Acoustofluidics-enhanced biosensing with simultaneously high sensitivity and speed [file 41378_2024_731_MOESM1_ESM.doc]

**Supporting Information**

Acoustofluidics-enhanced biosensing with simultaneously high sensitivity and speed

*Yuang Li^1,2^, Yang Zhao^1,*^, Yang Yang^3^, Wenchang Zhang^1,*^, Yun Zhang^1,2^, Sheng Sun^1,2^, Lingqian Zhang^1^, Mingxiao Li^1^, Hang Gao^1^, and Chengjun Huang^1,2,*^*

1. Institute of Microelectronics of the Chinese Academy of Sciences, Beijing, 100029, P. R. China

2. University of Chinese Academy of Sciences, Beijing, 101408, P. R. China

3. Department of Medicine, Brigham and Women’s Hospital, Harvard Medical School, Boston, MA, 02115, USA

***Corresponding Author:** huangchengjun@ime.ac.cn; [zhaoyang@ime.ac.cn](mailto:zhaoyang@ime.ac.cn); zhangwenchang@ime.ac.cn.

**Contents:**

1. Fabrication and testing of FIDT

2. Modification methods for PS microbeads and microchannels

3. Characterization and validation of surface modification methods

**Fabrication and testing of FIDT**

For the design and fabrication of FIDT, we used the standard photolithography and lift-off processes. As shown in Fig. S1, the lithium niobate substrate was cleaned, coated with a layer of photoresist, exposured with a pattern mask, developed to remove part of the photoresist to form the pattern, and deposited with metal. After that, we finally removed the photoresist to take away the unwanted metal to form the pattern of FIDT.


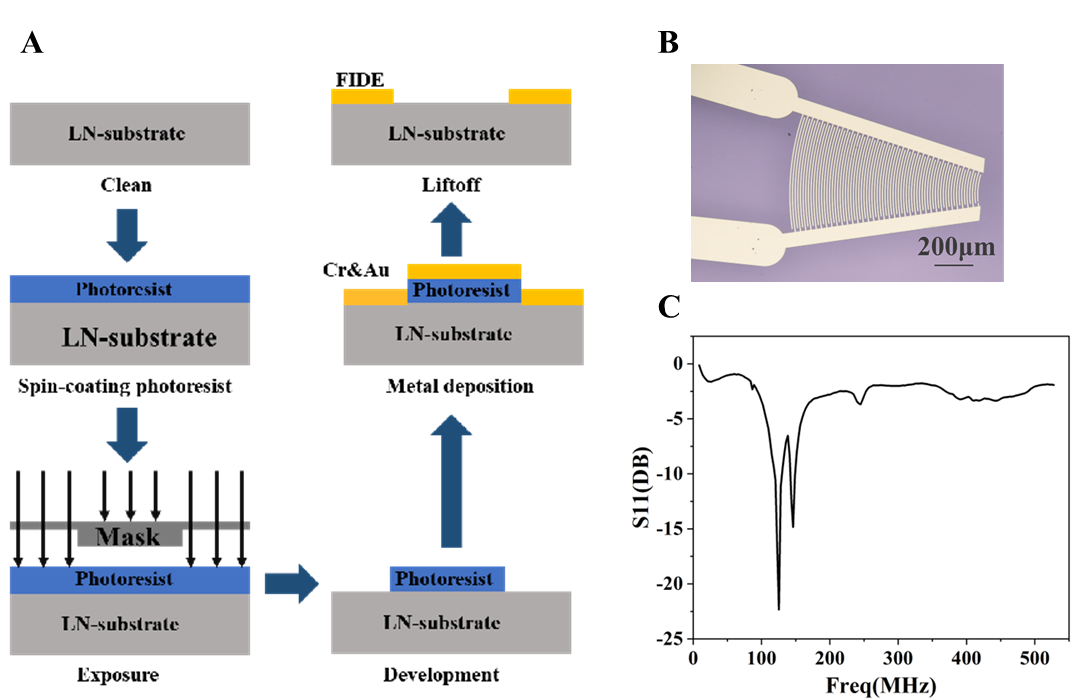


**Figure S1**. Fabrication process and characterization of FIDT.

**Modification methods for PS microbeads and microchannels**

The PS microbeads were washed with PBS and resuspended with MES ethane sulfonic acid solution after washing and centrifugation. PS microbeads were activated using MES-formulated EDC and NHS solutions. EDC can react with the carboxyl group on the surface of PS microbeads to form an amino-reactive O-acyl-urea intermediate, but the intermediate was unstable in aqueous solution and was easily hydrolyzed, so sulfo-NHS was added to convert the O-acylurea intermediate into an amino reactive NHS ester to accelerate the binding. The PS microbeads were mixed with the solution and shaken well. After centrifugation to remove the supernatant, the microbeads were resuspended in PBS. The PS microbeads were then mixed with the target solution and shaken to label the surface of the PS microbeads with the target molecules. After labeling, it was washed with PBS and resuspended by centrifugation. The PS microbeads containing the modified target molecules were then resuspended with 5% BSA in 1×PBS blocking solution to minimize the problem of nonspecific adsorption during subsequent detection. Finally, after centrifugation to remove the supernatant and resuspension with PBS, the modification of PS microbeads was completed.

After the microfluidic chip was fabricated, the channels were first cleaned with deionized water. Then, the surface silanol group (-SiOH) was converted to the amino group (-NH2) using APTES. The reagent was injected into the channel from the inlet, and the reaction was carried out after forming a self-assembled monolayer in the channel. The channel was then washed with deionized water. Then, 2.5% glutaraldehyde aqueous solution was injected into the channel, and the aldehyde group in the glutaraldehyde molecule was used to bind with the amino group on the surface of the device to realize the connection between APTES and the modified target, and the channel was washed with deionized water after the reaction. After that, the target reagent to be modified (antigen/antibody) was injected into the channel and reacted to immobilize the target molecule on the surface of the device. Then, the channels were blocked with 5% BSA in 1×PBS blocking solution to minimize the problem of nonspecific adsorption during subsequent detection. Finally, the channels were washed with PBS and can be stored at 4°C.

**Characterization and validation of surface modification methods**

To validate the modification method for microbeads and microchannels, we first performed the modification using a high concentration of fluorescent antibody. As shown in Fig. S2A/B, the fluorescent antibody was successfully modified to the surface of microbeads and microchannels. In addition, we modified the microbeads using a low concentration (100ng/mL) of antibody. The microbeads, after modification at different times, were analyzed, and the results are shown in Fig. S2C-D. The results showed that when the modification time was 20 minutes, the fluorescence was challenging to distinguish due to the low antibody concentration. Furthermore, when the modification time was two hours, the antibody fluorescence was weak, and when the time was increased to 24 hours, the fluorescence on the surface of microbeads could be clearly seen. This also suggests that the longer the modification time, the greater the chance and amount of antibody binding to the microsphere surface. The statistical results are shown in Fig. S2F. Moreover, as shown in Fig. S2G-i, when the antibody concentration is too low (0.1ng/mL), fluorescence analysis is no longer possible, and even if the modification time is long enough, fluorescence is still not observed.


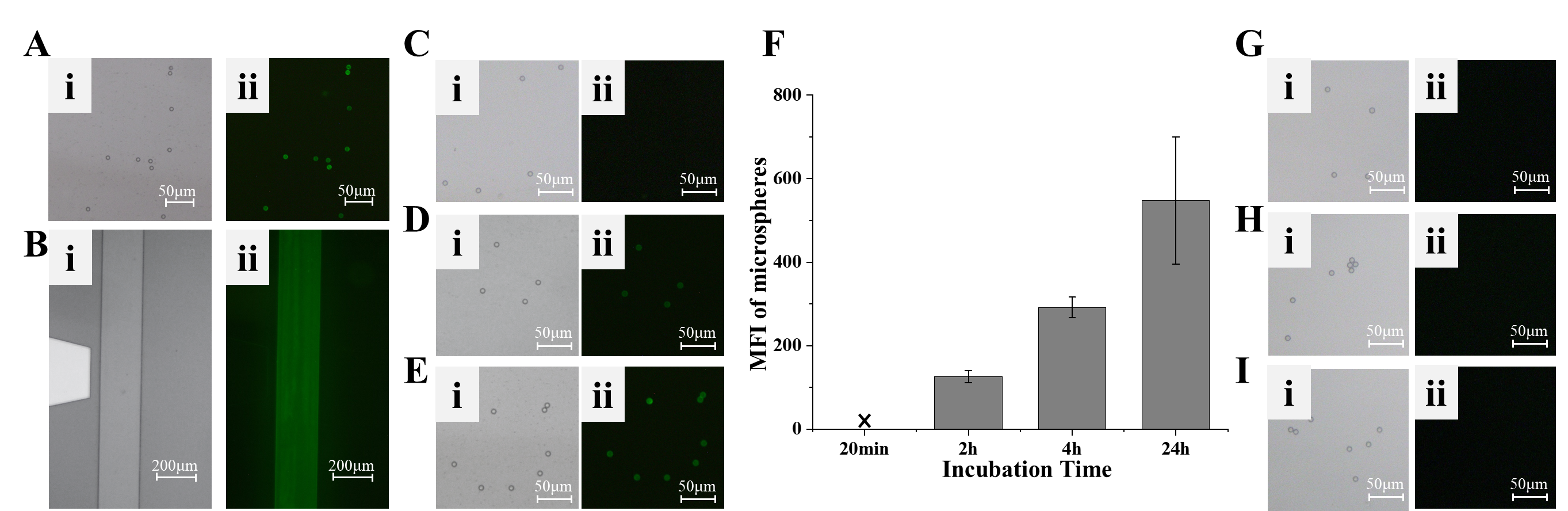


**Figure S2.** Fluorescence characterization and analysis of modification results. **A-B.** Results of microbeads and channels after fluorescent antibody modification. **C-E.** Bright-field and fluorescence pictures of microbeads modified with low concentration (100ng/mL) of fluorescent antibody for 20 minutes, 2 hours, and 24 hours, respectively. **F.** Statistical plot of modification time versus relative average fluorescence intensity. **G-I.** Bright-field and fluorescence images of microbeads were modified with low concentration (0.1ng/mL) fluorescent antibody for 20 minutes, 2 hours, and 24 hours.
